# Risk Factors for Postoperative Major Morbidity, Anastomotic Leakage, Re-Surgery and Mortality in Patients with Colonic Perforation

**DOI:** 10.3390/jcm13175220

**Published:** 2024-09-03

**Authors:** Maximilian Brunner, Lara Gärtner, Andreas Weiß, Klaus Weber, Axel Denz, Christian Krautz, Georg F. Weber, Robert Grützmann

**Affiliations:** Department of General and Visceral Surgery, Friedrich-Alexander-University Erlangen-Nürnberg (FAU), Krankenhausstraße 12, 91054 Erlangen, Germany; lara.gaertner@posteo.de (L.G.); andreas.weiss@uk-erlangen.de (A.W.); klaus.weber@uk-erlangen.de (K.W.); axel.denz@uk-erlangen.de (A.D.); christian.krautz@uk-erlangen.de (C.K.); georg.weber@uk-erlangen.de (G.F.W.); robert.gruetzmann@uk-erlangen.de (R.G.)

**Keywords:** colonic perforation, surgical management, risk factors, complications, mortality, re-surgery, anastomotic leakage

## Abstract

**Background/Objectives**: This study aimed to determine the risk factors associated with postoperative major morbidity, anastomotic/suture leakage, re-surgery and mortality in patients undergoing emergency surgery for colonic perforation. **Methods**: A total of 204 adult patients treated surgically for colonic perforation from 2016 to 2021 at the University Hospital Erlangen were included in a retrospective analysis. Patient demographics and pre-, intra- and postoperative parameters were obtained and evaluated among various outcome groups (in-hospital major morbidity, anastomotic/suture leakage, re-surgery and 90-day mortality). **Results**: Postoperative in-hospital major morbidity, anastomotic/suture leakage, need of re-surgery and 90-day mortality occurred in 45%, 12%, 25% and 12% of the included patients, respectively. Independent risk factors for in-hospital major morbidity were identified and included the presence of any comorbidity, a significantly reduced preoperative general condition, the localization of perforation in the right hemicolon and the need for an intraoperative blood transfusion. The only independent risk factor for anastomotic/suture leakage was the presence of any comorbidity, whereas no independent risk factors for re-surgery were found. An age > 65 years, a significantly reduced preoperative general condition and the need for an intraoperative blood transfusion were independent risk factors for 90-day mortality. **Conclusions**: Our study identified risk factors impacting postoperative outcomes in patients undergoing emergency surgery for colonic perforation. These patients should receive enhanced postoperative care and may benefit from individualized and targeted therapeutic approaches.

## 1. Introduction

Colonic perforations represent a life-threatening condition and are associated with persistently high morbidity and mortality rates, despite numerous advancements and innovations in surgical techniques and perioperative therapies over the past decades [1,2,3,4,5,6,7]. This condition can arise from various causes, most commonly diverticulitis. Other significant causes include malignancies, infections, inflammatory bowel disease, ischemia and iatrogenic factors, particularly during colonoscopy [8,9,10].

Leakage of colonic contents into the abdominal cavity can lead to intra-abdominal sepsis, potentially resulting in abscess formation, septic shock and multi-organ failure. Therefore, timely diagnosis and treatment are crucial and typically involve surgical intervention. Various surgical options are employed depending on the cause and severity of the colonic perforation. In some rare cases, perforation can be treated with suturing. However, the majority of patients require resection of the affected bowel segment, with three main reconstruction possibilities: blind closure of the distal bowel stump with an end colostomy (Hartmann procedure), anastomosis of the two bowel ends with a loop ileostomy or anastomosis of the two bowel ends without any stoma [3,11].

Identifying perioperative risk factors that can predict worse postoperative outcomes can enhance treatment quality using more personalized treatment approaches, intensified postoperative monitoring and tailored postoperative measures for high-risk patients.

The aim of this study was to determine the risk factors associated with the development of in-hospital major morbidity, anastomotic/suture leakage, re-surgery and 90-day mortality in patients receiving emergency surgery for colonic perforation.

## 2. Materials and Methods

In total, 204 patients who received emergency surgery for colonic perforation at the Surgical Department of the University Hospital of Erlangen—a high-volume center of colorectal surgery—between January 2016 and December 2021 were included in this retrospective analysis. Exclusion criteria were (1) age < 18 years, (2) absence of intraoperatively verified colonic perforation or (3) elective surgery.

The data that were collected included patient demographics, comorbidities, perforation characteristics, preoperative findings (blood results and radiological diagnostics), surgical details, microbiological findings and postoperative outcomes. The preoperative nutritional status of the patients was evaluated using the Nutritional Risk Score (NRS) [12]. An impaired preoperative general condition was considered present if any of the following criteria were met: presence of shock and/or the need for preoperative critical care or resuscitation. Time to surgery was defined as the interval between the documented time of the radiological examination that revealed the perforation and the start of the operation. Any deviation from the expected postoperative recovery was considered a postoperative complication and was categorized using the Clavien–Dindo classification [13]. Major morbidity was defined as grades III to V on the morbidity scale according to the Clavien–Dindo classification. Anastomotic/suture leakage included any possible postoperative bowel leakages such as anastomotic leakage, suture leakage and leakage of the Hartmann stump.

The primary aim of this investigation was to identify risk factors associated with in-hospital major morbidity, anastomotic/suture leakage, re-surgery and 90-day mortality after emergency surgical management for colonic perforation. Consequently, the study cohort was categorized based on the occurrence of the aforementioned four parameters.

This study received approval from the Ethical Committee of the University of Erlangen (23-194-Br, 6 June 2023).

### 2.1. Diagnostic Approach and Surgical Therapy

At the time of initial admission, all patients underwent blood tests, including a full blood count, creatinine and markers of inflammation. Preoperative assessments typically involved an abdominal ultrasound and an abdominal X-ray. If the diagnosis remained uncertain, a CT scan of the abdomen was performed.

All surgeries were performed either by a specialist in colorectal surgery or by an advanced resident under supervision of a colorectal surgery specialist. The choice of surgical procedure, including the type of resection and the decision to perform a stoma, was made by the operating surgeon and was recorded in the surgical report. Intraoperative samples for microbiological analysis were collected when deemed appropriate and based on the surgeon’s decision.

All patients received standardized postoperative antibiotic therapy with piperacillin and tazobactam. Based on the microbiological results from intraoperative swabs, the antibiotic therapy was adjusted accordingly. The duration of antibiotic therapy was determined by the surgeons overseeing the patient’s care on the ward (median in our cohort: 7 days).

### 2.2. Statistics

Data analysis was performed using SPSS (Version 28.0, IBM, Armonk, NY, USA). For comparisons of ordinal and continuous variables, the Mann–Whitney U test was employed, while categorical variables were analyzed with the chi-square test. A *p*-value of <0.05 was deemed statistically significant. Risk factors identified through univariate analysis for major morbidity, anastomotic/suture leakage, re-surgery, and mortality were further examined using multivariate analysis, and parameters with incomplete data were excluded. For continuous variables, the median was utilized as the cutoff point. Independent risk factors identified in the multivariate analysis were incorporated into the risk factor scoring system.

## 3. Results

### 3.1. Patient Cohort

A total of 204 patients who received emergency surgical management for colonic perforation during the study period were examined. The median age of the patients was 65 years, and 45% were female. Diverticulitis was the leading cause of colonic perforation, accounting for 44% of cases, followed by malignancy and inflammatory disease, each accounting for 8% of cases, and ischemia, which was responsible for 7% of cases. The majority of perforations were found in the sigmoid colon (62%), and other affected areas included the cecum (16%), ascending and descending colon (each 8%) and transverse colon (6%). A colon resection was required in 96% of all colonic perforations, and the most common was the Hartmann procedure (59%). Detailed demographic and perforation characteristics, preoperative blood test results, surgical information and microbiological findings are presented in Table 1.

### 3.2. Outcome Parameter after Surgery for Colonic Perforations

Out of 204 patients, 153 (75%) experienced postoperative complications, with 91 (45%) suffering from major morbidity (grades III to V according to the Clavien–Dindo classification). Anastomotic or suture leakage occurred in 12% of patients, 26% had a wound infection and 51 patients (25%) required a reoperation. The median hospital stay was 14 days. A total of 25 patients (12%) died during their hospital stay, and 26 (13%) died within the first 90 postoperative days. A total of 28 patients (13%) required readmission within the first 90 postoperative days (Table 2).

### 3.3. Risk Factors for Postoperative In-Hospital Major Morbidity

In the univariate analysis, ten risk factors were found to be significantly associated with the occurrence of in-hospital major morbidity: age > 65 years (*p* < 0.001), the presence of any comorbidity (*p* < 0.001), an impaired preoperative general condition (*p* < 0.001), a perforation of the right hemicolon (*p* < 0.001), a preoperative hemoglobin concentration ≤ 11.5 g/dL (*p* = 0.002), a preoperative creatinine concentration > 0.9 mg/dL (*p* < 0.001), a preoperative assessment of free abdominal air using radiology (*p* = 0.034), the need for a Hartmann procedure (*p* < 0.001), the need for an intraoperative blood transfusion (*p* < 0.001) and a positive intraoperative swab with at least two microorganisms (*p* = 0.034). Multivariate analysis revealed that the presence of any comorbidity (HR 3.4 (1.5–7.9), *p* = 0.004), an impaired preoperative general condition (HR 5.5 (1.5–19.9), *p* = 0.009), a perforation of the right hemicolon (HR 3.0 (1.4–6.6), *p* = 0.007) and the need for an intraoperative blood transfusion (HR 5.0 (2.1–12.0), *p* < 0.001) were independent risk factors for in-hospital major morbidity (Table 3).

### 3.4. Risk Factors for Anastomotic/Suture Leakage

In the univariate analysis, there were two parameters that were associated with the prevalence of an anastomotic/suture leakage: the presence of any comorbidity (*p* = 0.004) and a history of abdominal surgery (*p* = 0.048). In the multivariate analysis, only the presence of any comorbidity (HR 5.0 (1.4–17.5), *p* = 0.011) remained a significant risk factor (Table 3).

### 3.5. Risk Factors for Re-Surgery

In the univariate analysis, seven risk factors that indicated a necessity for re-surgery were identified: age > 65 years (*p* = 0.035), the presence of any comorbidity (*p* = 0.012), an impaired preoperative general condition (*p* = 0.037), a perforation of the right hemicolon (*p* = 0.008), a preoperative white blood cell count > 13.2 g/dL (*p* = 0.042), a preoperative creatinine concentration > 0.9 mg/dL (*p* = 0.013) and the need for an intraoperative blood transfusion (*p* = 0.006). None of these parameters reached significance in the multivariate analysis (Table 4).

### 3.6. Risk Factors for 90-Day Mortality

In the univariate analysis, nine risk factors were identified for 90-day mortality: age > 65 years (*p* = 0.011), an NRS ≥ 3 (*p* = 0.007), an impaired preoperative general condition (*p* < 0.001), a perforation of the right hemicolon (*p* = 0.002), a preoperative hemoglobin concentration ≤ 11.5 g/dL (*p* = 0.002), a preoperative creatinine concentration > 0.9 mg/dL (*p* = 0.027), a preoperative CRP value > 140 mg/L (*p* = 0.028), the need for a Hartmann procedure (*p* = 0.005) and the need for an intraoperative blood transfusion (*p* < 0.001). Of these identified risk factors, an NRS ≥ 3 (HR 4.8 (1.2–18.7), *p* = 0.023), an impaired preoperative general condition (HR 4.4 (1.4–13.7), *p* = 0.011) and the need for an intraoperative blood transfusion (HR 3.2 (1.2–9.1), *p* = 0.025) were independent risk factors in the multivariate analysis (Table 4).

### 3.7. Absolute Risk for Major Morbidity, Anastomotic/Suture Leakage and 90-Day Mortality

Table 5 presents the absolute risk values for in-hospital major morbidity, anastomotic/suture leakage and 90-day mortality based on the number of present independent risk factors.

## 4. Discussion

Colonic perforations are a severe emergency that demands immediate diagnosis and effective interventions. Identifying the risk factors associated with key outcome measures can enhance the quality of care by allowing for an ongoing refinement and customization of treatment strategies specifically designed for high-risk patients.

The demographic and perforation characteristics of our study cohort were largely consistent with those reported in other investigations on the surgical management of colonic perforations [1,2,3,4,5,6,7,11,14,15]. However, the differences in inclusion and outcome parameters can partially hinder the comparability of the data. In our study, we investigated colonic perforations from any origin and location within the entire colon, excluding the rectum, and focused on four key outcome parameters: in-hospital major morbidity, suture/anastomotic leakage, re-operation and 90-day mortality. Comparable studies in the literature are often limited to specific causes or locations within the colon or include the rectum and frequently focus solely on mortality as an outcome parameter [1,2,3,4,5,6,7,11,14,15].

The major morbidity rate in our cohort was 45%, which is a little bit higher than that reported in previous studies [4]. This discrepancy could be attributed to the worse ASA scores in our cohort. Comparable data about risk factors concerning morbidity are limited to overall morbidity, which affects comparability. Among the four identified risk factors for in-hospital major morbidity in our cohort, the need for intraoperative blood transfusion was already described by Lee et al. as an independent risk factor for morbidity [4]. Although the other three identified risk factors in our cohort are not explicitly described in the literature for colonic perforation, they are plausible since the presence of any comorbidity and an impaired preoperative general condition are well-known risk factors for morbidity in other surgical contexts [16]. Furthermore, a perforation of the right hemicolon was identified by Lee et al. as a risk factor for mortality, which aligns with our findings on major morbidity [4]. Other risk factors identified in the literature for morbidity, such as symptom duration, renal failure, fecal abdominal contamination, NLR (neutrophil-to-lymphocyte ratio) and PI (prognostic index) were not investigated in our study [6,7].

The occurrence of postoperative anastomotic/suture leakage and the need for re-surgery are parameters that have not been thoroughly investigated in prior research, resulting in a lack of comparable data. In our study cohort, the prevalence of anastomotic/suture leakage was 12%, which is slightly higher than that reported by Lee et al. [4]. The only identified risk factor of any comorbidity underscores the importance of preoperative health status for surgical outcomes. Re-surgery occurred in 25% of cases, included planned relaparotomies and was not associated with identifiable independent risk factors.

Our study also investigated 90-day mortality, which is a well-studied parameter due to its significant relevance. The mortality rate of 13% in our study falls within the range of previously published data (6.8–20.1%) [1,2,3,4,5,6,7]. Our analysis identified a Nutritional Risk Score (NRS) ≥ 3, a significantly reduced preoperative general condition and the need for intraoperative blood transfusion as risk factors for mortality. These factors are consistently described as prominent risk factors in the literature [2,4,6,7,17,18]. Other potential risk factors, such as time to surgery > 2 days, the occurrence of major morbidity, the presence of organ failures or renal failure, a worse ASA score, preoperative leucopenia, a right colon perforation, diffuse peritonitis, and an elevated PLR (platelet-to-lymphocyte ratio) were either not confirmed in our study or not investigated [2,3,4,5,6,7].

The identified risk factors for surgical outcomes following the surgical management of colonic perforation provide valuable insights for improving patient care. By recognizing these risk factors, targeted preventive measures can be implemented for high-risk patients. For instance, patients with elevated risk profiles could benefit from pre-, intra- and postoperative optimization strategies, such as pre- and intraoperative blood management, nutritional support, enhanced antibiotic prophylaxis or more aggressive management of comorbid conditions. Additionally, more extensive postoperative monitoring could be employed to detect complications earlier, allowing for a timely intervention and potentially reducing the severity of adverse outcomes.

Despite these potential benefits, our study has notable limitations that must be addressed. The retrospective design and single-center nature of the study introduce the possibility of biases, which could affect the generalizability of the findings. Furthermore, the heterogeneity in the causes of colonic perforations and the variability in surgical techniques and expertise among the surgeons may impact the consistency of the results. To mitigate these limitations, future research should aim for multicenter, prospective studies with standardized protocols and a focus on minimizing variability in surgical practices. Such approaches will enhance the reliability of the data and provide more robust evidence for optimizing management strategies for colonic perforations.

## 5. Conclusions

In the surgical management of colonic perforations, a precise risk assessment is essential for identifying patients at higher risk of poor outcomes. By utilizing risk classification, efforts can be focused on and postoperative care can be tailored to the specific needs of high-risk individuals, thereby enhancing the likelihood of favorable outcomes through personalized therapeutic strategies.

## Figures and Tables

**Table 1:** Characteristics of patients who underwent emergency surgery for colonic perforation.

**Demographic data**	**Number**	204
	**Age (years), median (IQR)**	65 (18)
	**Gender, *n* (%)**	
	**Female**	91 (45)
	**Male**	113 (55)
	**ASA, *n* (%)**	
	**I**	7 (3)
	**II**	57 (28)
	**III**	91 (45)
	**IV**	43 (21)
	**V**	2 (1)
	**Unknown**	4 (2)
	**Body mass index (BMI) (kg/m^2^), median (IQR)**	26.4 (7.9)
	**Nutritional risk score (NRS), *n* (%)**	
	**<3**	106 (52)
	**≥3**	98 (48)
	**Comorbidity, *n* (%)**	
	**Hypertension**	116 (57)
	**Coronary heart disease**	33 (16)
	**Diabetes**	30 (15)
	**Heart insufficiency**	23 (11)
	**Chronic renal insufficiency**	19 (9)
	**Chronic obstructive pulmonary disease (COPD)**	18 (9)
	**Smoking, *n* (%)**	62 (30)
	**Previous abdominal surgery, *n* (%)**	81 (40)
	**Preoperative steroids/immunosuppression, *n* (%)**	37 (18)
**Perforation characteristics**	**Preoperative general condition, *n* (%)**	
	**Well or slightly reduced**	175 (86)
	Impaired *	29 (14)
	**Etiology of perforation, *n* (%)**	
	**Diverticulitis**	89 (44)
	**Malignancy**	16 (8)
	**Inflammatory disease**	17 (8)
	**Ischemia**	14 (7)
	**Others (idiopathic, traumatic, another disease)**	21 (10)
	**Unknown**	47 (23)
	**Localization of perforation, *n* (%)**	
	**Cecum**	32 (16)
	**Ascending colon**	16 (8)
	**Transverse colon**	13 (6)
	**Descending colon**	16 (8)
	**Sigmoid colon**	127 (62)
**Preoperative blood results**	Preoperative white blood cell count (WBC) (10^9^/L) (*n* = 142) **, median (IQR)	13.2 (8.6)
	Preoperative hemoglobin (g/dL) (*n* = 142) **, median (IQR)	11.5 (3.9)
	Preoperative creatinine (mg/dL) (*n* = 164) **, median (IQR)	0.9 (0.9)
	Preoperative C-reactive protein (CRP) (mg/L) (*n* = 166) **, median (IQR)	140 (178)
**Radiological diagnostic**	**Free abdominal air, *n* (%)**	108 (53)
**Surgical details**	**Kind of procedure, *n* (%)**	
	**Suturing**	8 (4)
	**Resection**	196 (96)
	Surgical reconstruction (*n* = 196) ***, n (%)	
	**Anastomosis without stomata**	45 (23)
	**Anastomosis with protective stomata**	36 (18)
	**Hartmann procedure**	115 (59)
	**Surgical approach, *n* (%)**	
	**Open**	185 (91)
	**Laparoscopic**	11 (5)
	**Conversion from laparoscopic to open**	8 (4)
	**Surgeon’s expertise, *n* (%)**	
	**Advanced resident**	77 (38)
	**Specialist in colorectal surgery**	127 (62)
	**Time to surgery (h), median (IQR)**	4 (5)
	**Operative time (min), median (IQR)**	169 (66)
	**Intraoperative blood transfusion, *n* (%)**	53 (26)
	**Intraoperative blood loss (mL), median (IQR)**	200 (200)
**Microbiology**	**Intraoperative swab, *n* (%)**	
	**≤1 microorganism**	91 (45)
	**≥2 microorganisms**	103 (55)

ASA = American Society of Anesthesiologists score; IQR = Interquartile range. * indicates presence of shock and/or requirement for preoperative critical care or reanimation; ** indicates missing data; *** indicates only patients with resection.

**Table 2:** Outcome parameter for patients who underwent emergency surgery for colonic perforation.

Time	Outcome Parameter	*n* (%)
**In-hospital**	**Clavien–Dindo, *n* (%)**	
	**0**	51 (25)
	**I**	16 (8)
	**II**	46 (23)
	**III**	40 (20)
	**IV**	26 (13)
	**V (Mortality)**	25 (12)
	**Anastomotic/suture leakage, *n* (%)**	25 (12)
	**Wound healing disorder, *n* (%)**	53 (26)
	**Re-surgery, *n* (%)**	51 (25)
	Duration of postoperative hospital stay (in days) (*n* = 179) *, median (IQR)	14 (17)
**After discharge**	90-day readmission (n = 179) *, *n* (%)	28 (16)
	**90-day mortality, *n* (%)**	26 (13)

IQR = Interquartile range. * indicates exclusion of patients with postoperative in-hospital mortality.

**Table 3:** Risk factor analysis for in-hospital major morbidity and anastomotic/suture leakage in patients following emergency surgery for colonic perforation.

	In-Hospital Major Morbidity (*n* = 91)				Anastomotic/Suture Leakage (*n* = 25)			
	Univariate	Multivariate			Univariate	Multivariate		
		HR	95% CI	*p*		HR	95% CI	*p*
**Age (** **≤65 vs. >65 years)**	**<0.001**	1.7	0.8–3.5	0.162	0.525	-	-	-
**Gender (female vs. male)**	0.325	-	-	-	1.000	-	-	-
**BMI (** **≤25 vs. >25 kg/m^2^)**	0.775	-	-	-	0.134	-	-	-
**NRS (<3 vs. ≥3)**	0.260	-	-	-	0.522	-	-	-
**Any comorbidity (no vs. yes)**	**<0.001**	**3.4**	**1.5–7.9**	**0.004**	**0.004**	**5.0**	**1.4–17.5**	**0.011**
**Smoking (no vs. yes)**	0.541	-	-	-	0.353	-	-	-
**Previous abdominal surgery (no vs. yes)**	0.666	-	-	-	**0.048**	0.4	0.1–1.1	0.067
**Preoperative steroids/immunosuppression (no vs. yes)**	0.143	-	-	-	0.265	-	-	-
**Significantly reduced preoperative general condition (no vs. yes)**	**<0.001**	**5.5**	**1.5–19.9**	**0.009**	0.541	-	-	-
**Localization of perforation (right hemicolon vs. left hemicolon)**	**<0.001**	**0.3**	**0.2–0.7**	**0.007**	0.108	-	-	-
**Preoperative WBC (≤13.2 vs. >13.2 × 10^9^/L)**	0.314	-	-	-	0.084	-	-	-
**Preoperative hemoglobin (≤11.5 vs. >11.5 g/dL)**	**0.002**	*	*	*	0.626	-	-	-
**Preoperative creatinine (≤0.9 vs. >0.9 mg/dL)**	**<0.001**	*	*	*	0.172	-	-	-
**Preoperative CRP (≤140 vs. >140 mg/L)**	0.351	-	-	-	0.652	-	-	-
**Free abdominal air (no vs. yes)**	**0.034**	1.1	0.5–2.4	0.761	0.832	-	-	-
**Kind of surgery (Hartmann procedure vs. other)**	**<0.001**	0.8	0.3–1.7	0.516	0.830	-	-	-
**Surgeon’s expertise (resident vs. specialist)**	0.772	-	-	-	1.000	-	-	-
**Time to surgery (≤4 vs. >4 h)**	0.055	-	-	-	0.087	-	-	-
**Operative time (≤169 vs. >169 min)**	0.887	-	-	-	0.523	-	-	-
**Need of intraoperative blood transfusion (no vs. yes)**	**<0.001**	**5.0**	**2.1–12.0**	**<0.001**	0.810	-	-	-
**Intraoperative swab (≤1 microorganism vs. ≥2 microorganisms)**	**0.034**	2.1	1.0–4.5	0.058	0.672	-	-	-

Bold = significant; BMI = Body mass index; WBC = White blood cell count; CRP = C-reactive protein. * indicates exclusion from multivariate analysis due to missing data.

**Table 4:** Risk factor analysis for the need for re-surgery and 90-day mortality in patients following emergency surgery for colonic perforation.

	Re-Surgery (*n* = 51)				90-Day Mortality (*n* = 26)			
	Univariate	Multivariate			Univariate	Multivariate		
		HR	95% CI	*p*		HR	95% CI	*p*
**Age (** **≤65 vs. >65 years)**	**0.035**	1.5	0.7–3.0	0.311	**0.011**	1.3	0.4–4.5	0.683
**Gender (female vs. male)**	0.517	-	-	-	0.836	-	-	-
**BMI (** **≤25 vs. >25 kg/m^2^)**	0.142	-	-	-	0.060	-	-	-
**NRS (<3 vs. ≥3)**	0.746	-	-	-	**0.007**	**4.8**	**1.2–18.7**	**0.023**
**Any comorbidity (no vs. yes)**	**0.012**	2.1	0.9–4.7	0.072	0.204	-	-	-
**Smoking (no vs. yes)**	1.000	-	-	-	0.496	-	-	-
**Previous abdominal surgery (no vs. yes)**	0.742	-	-	-	0.286	-	-	-
**Preoperative steroids/immunosuppression (no vs. yes)**	0.529	-	-	-	0.099	-	-	-
**Significantly reduced preoperative general condition (no vs. yes)**	**0.037**	1.4	0.5–3.5	0.506	**<0.001**	**4.4**	**1.4–13.7**	**0.011**
**Localization of perforation (right hemicolon vs. left hemicolon)**	**0.008**	0.5	0.2–1.0	0.054	**0.002**	0.4	0.1–1.1	0.086
**Preoperative WBC (≤13.2 vs. >13.2 × 10^9^/L)**	**0.042**	*	*	*	1.000	-	-	-
**Preoperative hemoglobin (≤11.5 vs. >11.5 g/dL)**	0.135	-	-	-	**0.002**	*	*	*
**Preoperative creatinine (≤0.9 vs. >0.9 mg/dL)**	**0.013**	*	*	*	**0.027**	*	*	*
**Preoperative CRP (≤140 vs. >140 mg/L)**	0.483	-	-	-	**0.028**	*	*	*
**Free abdominal air using radiology (no vs. yes)**	0.627	-	-	-	0.404	-	-	-
**Kind of surgery (Hartmann procedure vs. other)**	0.051	-	-	-	**0.005**	0.3	0.1–1.0	0.057
**Surgeon’s expertise (resident vs. specialist)**	0.740	-	-	-	1.000	-	-	-
**Time to surgery (≤4 vs. >4 h)**	0.354	-	-	-	0.818	-	-	-
**Operative time (≤169 vs. >169 min)**	0.627	-	-	-	0.832	-	-	-
**Need of intraoperative blood transfusion (no vs. yes)**	**0.006**	1.9	0.9–4.0	0.088	**<0.001**	**3.2**	**1.2–9.1**	**0.025**
**Intraoperative swab (≤1 microorganism vs. ≥2 microorganisms)**	0.074	-	-	-	0.059	-	-	-

Bold = significant; BMI = Body mass index; WBC = White blood cell count; CRP = C-reactive protein. * indicates exclusion from multivariate analysis due to missing data.

**Table 5:** Risk for morbidity, anastomotic/suture leakage and 90-day mortality according to the number of identified independent risk factors.

Number of Risk Factors	In-Hospital Major Morbidity *		Anastomotic/Suture Leakage **		90-Day Mortality ***	
	n	Risk	n	Risk	n	Risk
**0**	50	0.0%	79	3.8%	77	0.0%
**1**	75	28.6%	125	17.6%	80	8.8%
**2**	53	67.9%			41	41.5%
**3**	17	88.2%			6	33.3%
**4**	9	100%				

* four independent risk factors for morbidity: presence of any comorbidity, impaired preoperative general condition, a perforation of the right hemicolon and the need for an intraoperative blood transfusion. ** one independent risk factor for anastomotic/suture leakage: presence of any comorbidity. *** three independent risk factors for 90-day mortality: NRS ≥ 3, impaired preoperative general condition and the need for an intraoperative blood transfusion.

## Data Availability

All data are included in the manuscript and the tables.
